# Do Athletes Have More of a Cognitive Profile with ADHD Criteria than Non-Athletes?

**DOI:** 10.3390/sports9050061

**Published:** 2021-05-11

**Authors:** Elizabeth Ekman, Arto Hiltunen, Henrik Gustafsson

**Affiliations:** 1 Department of Social and Psychological Studies, Faculty of Arts and Social Sciences, Karlstad University, 65188 Karlstad, Sweden; elizabeth.ekman@gmail.com (E.E.); a.j.hiltunen@outlook.com (A.H.); 2 Department of Educational Studies, Faculty of Arts and Social Sciences, Karlstad University, 65188 Karlstad, Sweden; 3 Department of Sport and Social Sciences, Norwegian School of Sport Sciences, Postboks 4014 Ullevål Stadion, 0806 Oslo, Norway

**Keywords:** attention, concentration, dual careers, elite sports, performance, school, talent

## Abstract

The current study investigates the possibility that athletes have more parallel ADHD symptoms than non-athletes. High-level youth sport athletes were compared with non-athletes in leisure time (i.e., sport) and in the school in ADHD symptoms. Athletes and students were evaluated by a trained psychotherapist using Adult ADHD Self-Report Scale (ASRS) on activities at school and during activities in leisure/sports time. They also filled in the Autism Spectrum Questionnaire (AQ) as a self-report assessment. Results showed significant differences in ASRS-scores for athletes in school and in their sport, with high scores in school and low scores in sport. No differences were found in AQ between the groups. The findings indicate that many athletes might display a cognitive profile of parallel of ADHD criteria. Future research needs to further investigate potential benefits of the cognitive profile in athletes and how they handle different contexts including sport and school settings.

## 1. Introduction

Attention deficit hyperactivity disorder (ADHD) is considered a disorder that can cause behavioral and emotional problems for the individual [1]. Studies have shown how the environment, as well as physical activity, can influence ADHD in a positive direction and prevent its negative effects [1,2]. Most ADHD studies focus on the problems the individual has developed due to the disorder. For example, motor problems, struggling socially and academically in youth and in adulthood problems with unemployment, education and social functioning [3,4,5]. In sport research, focus has been on problems with aggression and higher prevalence of concussion [6,7,8]. Less focus has been on how ADHD can be used to advantage for the individual when the cognitive profile, (i.e., hyper focusing, high energy [9,10] and environmental factors (e.g., structure) are considered and interact [11]).

Attention deficit hyperactivity disorder involves distractibility, and difficulty with attention, impulse control, and activity control, in reference to what the situation requires [12]. For an individual to meet the Diagnostic and Statistical Manual of Mental Disorders (DSM)-V criteria (Inattention and hyperactivity are considered core symptoms of ADHD) [12], they need to cause impairment and be exhibited, in two different settings, for example at home and at school or work [13]. Studies show that the ADHD symptoms are part of a spectrum, which ranges from hyperactivity/impulsivity to attentional difficulties, and genetic studies have proposed that ADHD should be regarded as a set of behavioral traits that are also present in the general population but in a less extreme way [1,2]. In addition to the core symptoms of inattention and hyperactivity, one of the main deficits in ADHD, is “selective attention,” i.e., selecting a target item while attenuating irrelevant stimuli in the presence of conflicting, distracting information [14,15]. This extreme form of attention or “hyper focusing” is however, not discussed in current conceptions of ADHD symptoms. Rather the emphasis is on problems involving symptoms like inattention. Hyper focusing is most likely to occur in situations where the individual is goal-oriented and receives immediate feedback from the activity in progress, i.e., the individual finds the activity stimulating and shuts out irrelevant information [16,17].

An interesting theory is Hartmans anthropological/sociological theory where ADHD is described as hunter in a farmer society. In this model, it is stated that ADHD traits of hunter–gatherers would have been environmentally advantageous. This includes hyperactivity, thus being energetic and tireless behaviour, and having high impulsivity, and the ability to change the strategy quickly. Accordingly, the very attributes that render ADHD individuals good “hunters” (e.g., constant monitoring of environment, flexibility, being able to throw themselves into a chase on a moment’s notice), are less compatible with modern, daily demands of the “farmer society.” The potentially advantageous traits from hunters are easily translated into sports where being energetic, monitoring the field of play and being ready to take action are effective in many situations and types of sports.

Difficulties in focusing and keeping attention have been well studied in ADHD. Another area that has been researched is selective attention although few studies have considered how selective attention can be an advantage, i.e., in terms of hyper focusing, which has been reported as a phenomenon of ADHD [10,18]. Hyperfocusing has not previously been defined in scientific research or examined as a symptom of ADHD, but has been suggested as a separate dimension of adult ADHD [10,19]. The ability to focus has in sport been considered to be key for performance [20] and for this reason “hyper focusing” can be an asset in sport, and may even be crucial for high performance [21]. In light of this knowledge, inattention and hyperactivity as well as selective attention do not necessarily have to be problematic symptoms for an individual with ADHD traits, but can be regarded in terms of hyper focusing. There are studies showing that ADHD children are more active and involved in physical activity compared to non-ADHD children [22]. For athletes, their activity e.g., sport, not only involves physical activity but also motivation, stimulation and reward as well as structure, routine and social interaction. These are attributes that in previous studies have been shown to have a positive influence on ADHD symptoms [23,24]. In adolescence, physical activity has been reported to improve the ADHD symptoms in the individual as well as his or her academic performance and the adolescence overall attitude [1,24,25]. It has been shown that physical activity increases levels of dopamine and norepinephrine, which are deficient in ADHD children and adolescents [26]. Several studies suggest that physical exercise also reduces stress, negative affect, anxiety, and depression, as well as behavior problems, poor impulse control, and inattentiveness, and is therefore beneficial to individuals with ADHD [1,21]. Studies have also suggested that children with ADHD, need formal structure, supervision and guidance in sports activities [27], and that sustained involvement in structured physical activity benefits motor, cognitive, social, and behavioral functioning in these children [25]. Sports activities could, in other words, be considered not only to involve physical activity that stimulates neurotransmitters, but also to give reward and stimulation, as well as structure and routine, and consequently have a positive effect on school performance [26].

Furthermore, studies have shown that reward, in neurofeedback, has a positive influence on ADHD and, furthermore, a positive effect on endurance, which is a difficulty for ADHD children [14]. In sport, the involvement and engagement in vigorous physical activity, has shown itself to be a reinforcer as it is concurrently praised [24]. Therefore, the stimulation, the structured activity, and the continuous reward that sports participation provides, are likely to have a positive effect on the ADHD profile. This has been confirmed in a study where intensive sports participation was shown to be negatively related to anxiety and depression in children with ADHD [28]. In addition, participation in sports activities provides opportunities for social skills training, which can result in increased social skills, self-awareness, and social status, in both ADHD and non-ADHD youths [29]. It decreases the negative ADHD symptoms and improves the personal development, cognitively and emotionally [30], as well as providing better social status and increasing the person’s social skills, which results in less problematic behavior [31,32,33]. Accordingly, sports participation influences not only physical development, but also social, cognitive, and emotional development in the individual with ADHD.

Research in ADHD in athletes has mainly focused on the negative effects of ADHD. This includes studies reporting an increased risk of injury [8], and higher levels of aggression and emotional reactivity [7]. As mentioned, there might be several positive aspects of ADHD symptoms in the sport context. Early on, Hallowell and Ratey took a positive view based on their clinical observations (outside sport) [34]. Though they admit that there is no evidence for an “attention deficit disorder (ADD) personality” and “ADD personality traits,” they stress the importance of recognizing the following abilities and attributes in individuals with ADHD: high energy, creativity, intuitiveness, resourcefulness, tenacity, hardworkingness, a never-say-die approach, warm-heartedness, a trusting and forgiving attitude, sensitivity, the ability to take risks, flexibility, and a good sense of humor [34]. Many of these attributes are beneficial in sports, including hardworkingness and high energy [35], which are essential in order to handle the high training load required to become a high-level athlete [36,37].

Interestingly, it has been suggested that ADHD may be more common in competitive athletes than in the general population [22,38]. In a systematic review including 17 studies, the prevalence of ADHD varied between 4.2% and 14.1% in young athletes [39]. This can be compared with an estimated prevalence of ADHD in the population ranging from 5% to 15% in the general population [40]. Thus on youth athletes there does not seem to higher prevalence. However, in adult elite athletes a prevalence of 7% to 8% has been found, compared with the general population 0.8% to 2.4% [22,41]. Albeit not fully known, the reason ADHD may be more common in athletes than in the general population could be due to the positive reinforcing and attentional activating effects of physical activity including decreased symptoms and an arena to excel [22,42]. Thus, children will find exercise rewarding and be drawn to sport.

In conclusion, some common symptoms of ADHD may enhance athletic performance. Many children with ADHD report to ‘hyperfocus’ on their enjoyable sporting activities without being distracted [43]. It has also been suggested that the natural impulsivity athletes with ADHD have may provide an advantage in sports that require quick decision making and problem solving [41]. However, positive effects of ADHD in athletes have not to a large extent been systematically studied, and research on a cognitive profile with ADHD criteria-like symptoms in athletes needs further examination. Few studies compare athletes with ADHD to matched controls, and less attention has been given to the potential differences in experiencing ADHD criteria in different contexts such as leisure activities and school.

The aim of the current study was to compare athletes to non-athletes, both of school-going age, in terms of Adult ADHD Self-Report Scale (ASRS) criteria [44], i.e., ADHD symptoms, in leisure time and at school. Leisure time for the athletes was spent on their main sports activity.

## 2. Materials and Methods

### 2.1. Participants

A total of 200 students, 112 female and 88 male (*M*age 16.94 years, age range 16–19 years), were recruited from two high schools in Sweden, one group being athletes (40 women, 69 men, *M*age 16.64; SD 1.79) and another, non-athletes (48 women, 43 men, *M*age 17.23; SD 0.79). The athlete group were enrolled in the Swedish national sports talent program (Nationell Idrottsutbildning, NIU) and intensively involved in their sport and are considered among the most talented in the country and are not having any diagnosis nor do they have disability service at the sport academies. They competed at club/local (11.2%), regional (23.4%), national (52.3%), and international (13.1%) level and represented a variety of sports including team sports (e.g., ice hockey and football: 79.8%) and individual sports (e.g., track and field, and tennis: 20.2%). The athletes invested 12.20 (standard deviation (SD) 4.46; range 2–24) hours per week in training and competition and had participated in sport for a median of 9.29 (SD 2.85; 1–14) years. Both groups were matched in age and school programs, thus studying at the same theoretical educational program.

### 2.2. Measurements

The most common and initial instrument used to identify ADHD is the ASRS [44]. It was developed by the World Health Organization (WHO) for self-assessment of ADHD symptoms. The first six questions in the ASRS can be used as a short version of the instrument, ASRS Screening (ASRS-S) [45]. The ASRS has been designed in accordance with DSM-IV criteria for inattention and hyperactivity and impulsivity for ADHD. There are 18 items and the response options range from 0 = never, 1 = rarely, 2 = sometimes, 3 = often, to 4 = very often. The possible total ranges from 0 to 72 [45].

The ASRS-S is a very good tool for clinical evaluation of ADHD, if made by a trained and experienced clinical interviewer and not as a self-report data [46]. The three-stratum version of the scale has shown good concordance with blind clinical diagnoses and predicted probabilities of clinical diagnoses in the general population [44]. One question in the ASRS and ARSR-S relates to hyperactivity, a core symptom in ADHD [47], with children with ADHD reported to have a higher level of activity compared to typical children [33]. The Swedish version is validated for adolescents [46].

The Autism Spectrum Quotient (AQ) was selected because several studies have shown that Autism Spectrum Disorder (ASD) symptoms coexist in individuals with ADHD [48], especially in the core domains of ASD, such as social interaction, communication, and repetitive behaviors, and that these symptoms may contribute to greater stability of ADHD symptoms [49]. Greater ADHD symptoms appear to be associated with greater ASD symptoms. It has shown that especially in core domains of ASD, such as social interaction, communication and repetitive behaviors that these symptoms may contribute to greater stability of ADHD symptoms [49].

The AQ is one of the most widely used measures of ASD and has been found to be predictive of diagnosis of ASD in a clinical setting [50]. Questions in the AQ are associated with autism spectrum and cover social skills, communications skills, imagination, attention to detail, and attention switching/tolerance of change. There are 50 statements, and 1 point is scored for each answer that gives any indication of ASD, according to behaviors considered being core symptoms for ASD [50,51]. In a study by Baron–Cohen et al., a score of ≥32 is reported to indicate “clinically significant levels of autistic traits,” while the control group is reported to have scored 16.2 [51]. AQ has been validated in Swedish and function as an index of ASD [50].

### 2.3. Procedure

The Regional Ethics Review Board in Uppsala (Dnr. 2016/451) approved the study and after informed consent participants were interviewed by a trained psychotherapist with long experience and good knowledge of ADHD and autism spectrum disorder (ASD). In the interview, they were estimated on ASRS regarding school activity and leisure time/their sport activity time. The athlete themselves also filled in the AQ. The instruments were collected by the interviewer at the end of the interview. All text on the instruments about diagnoses was excluded and all questions were answered without mentioning of ADHD and ASD. The purpose of the evaluations was to identify parallel criteria between the two instruments, and not to identify a diagnosis. The instruments were named 1 and 2, in order for the students to be less influenced by their own preconceived opinion about diagnoses.

### 2.4. Statistical Analysis

Data analysis was carried out to compare the two groups in terms of ASRS criteria, in leisure time and in the school environment. The processing of data was conducted using IBM SPSS Statistics for Windows, Version 24.0. (IBM Corp.: Armonk, NY, USA) A two-way analysis of variance (ANOVA) F-test was used to analyse differences between the groups (athletes vs. non-athletes) and pair-wise post-hoc comparisons with Tukey’s HSD test was used to compare the sport athletes during sports activities and school. Data were screened for normality and homogeneity of variances was tested using the Levene statistic.

## 3. Results

### 3.1. Descriptive Analyis

Means and standard deviation are shown in Table 1. No significant differences were found in the analysis of AQ scores of the athletes (M = 14.30, SE = 0.40) compared to controls (M = 14.24, SE = 0.52). Further, there were no significant (*p* > 0.05) differences between club/local, regional, national, or international levels, in terms of hours invested in training and competitions.

### 3.2. Main Analysis

Two-way ANOVA (split-plot design) showed statistically significant between-group differences in ADHD criteria (F(1,195) = 157.3, *p* < 0.001), as well as within-group differences in school- and leisure activities (F(1,195) = 335.7, *p* < 0.001). Also, the interaction term for ADHD criteria vs. school- and leisure/sport activities was significant (F(1,195) = 365.9, *p* < 0.001).

**Figure 1:**
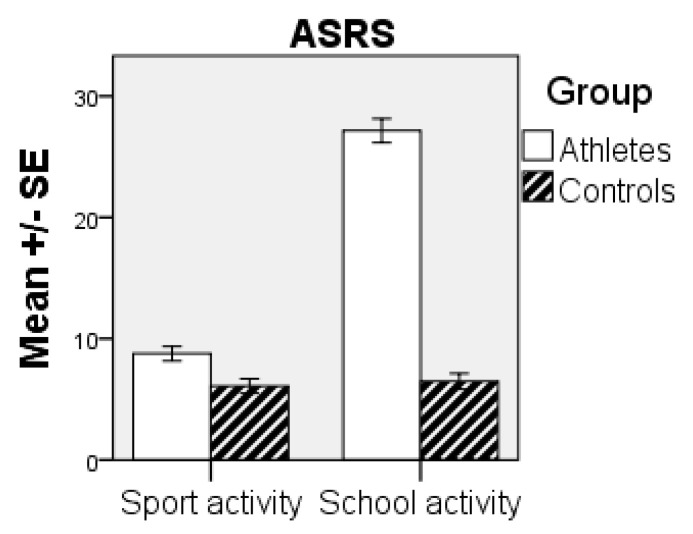
Comparison between groups in ADHD symptoms. SE = standard error. ASRS = Adult ADHD Self-Report Scale.

Pair-wise post-hoc comparisons with Tukey’s HSD test showed the significant (*p* < 0.001) difference between the groups during school activities, but not during leisure/sport activities (*p* > 0.05), with higher scores for the athlete group (see Figure 1). Further, comparison between the activities in the athlete group showed statistically significant (*p* < 0.001) differences between the activities, i.e., leisure/sports activities and school activities [Cohen’s d = 2.19].

## 4. Discussion

In this study, we have focused on how an ADHD profile is common among athletes, and have demonstrated significant differences in ASRS criteria for athletes during school vs. leisure time, i.e., time spent on their sport activities. They scored high on the ASRS with regard to their school activity and the school environment, and had low scores in their sports activity. The within-group effect size between the activities (sports and school activities in the group of athletes) was large [52]. For the control group, the scores were low for both school and leisure activities and significantly lower than the athletes in school. These findings are interesting and as suggested by others, in the athlete group, sport activities offer a structured and stimulating atmosphere thereby influencing the cognitive profile of ADHD [22]. The ADHD “symptoms” can thus be seen as a profile with functional behaviours, as well as emotional and cognitive strengths (e.g., endurance and goal orientation) with the ability to “hyper focus”.

In Hartmann’s sociological/anthropological theory, the ADHD cognitive profile is described as a hunter in a farming society [53] and based on the hypothesis that the traits associated with ADHD are better for hunters–gatherers and worse as settlers. Hartmann describes the ADHD individual as an expert on hunting with ability to hyper focus and at the same time scanning the environment, ready to take action (i.e., perceive information from attended channels while also doing so from unattended channels) [14,15,53]. Hartmann’s theory describes this as having an attentional advantage in the nomadic society, an advantage possibly also in a sport context and could easily be translated into describing for example a soccer player, highly active in a football match. Thus, the findings support the suggestions that certain aspects of ADHD might be beneficial in a sport context, as long as the activity is interesting and stimulating [22]. In the school environment, the activity might not be as stimulating and not as clearly goal-oriented, which might explain the high score on the ARSR regarding the school activity of the athlete group.

Several studies indicate that physical activity and environmental factors have a positive effect on inattention and hyperactivity, which are considered core symptoms of ADHD [47], and also have a positive effect on stress, negative affect, anxiety, and depression, as well as decreasing bad conduct and poor impulse control [54]. Physical activity appears to improve all ADHD symptoms, and can improve individuals’ overall attitude and personal development, cognitively and emotionally, as well as academic performance [1,2]. For athletes, sport is motivating, stimulating, and rewarding and involves physical activity, structure, routine, and social interaction. Being active in sport means benefiting from rewards and stimulation as well as structure and routine.

Children/adolescents involved in sport often demonstrate discipline and commitment [55], and evidence suggests that these traits carry over into school and other situations in the community [55,56]. All these findings are evidence for an environmental influence in children with ADHD [11,57,58,59] and that the brain development is highly responsive, not only to increased levels of physical activity/exercise, but also to environmental enrichment for cognitive development [53].

Based on the discussion above, a question for future research would be whether we can learn from these studies and, instead of over diagnosing and overtreating children/adults [48,60], look at the environmental factors and influence these before a diagnosis is considered. This will probably not reduce all problems, but it might decrease some of them and help us to better see the advantage of these children’s traits. When ADHD appeared in the second edition of the Diagnostic and Statistical Manual for Mental Disorders [61], hyperactivity was not considered an essential aspect of the disorder. The criteria that define ADHD have changed over time [62]. A question of interest would be whether this continuous change will continue. In 1970, the term “attention deficit disorder (ADD)” was used; today, the condition is named ADHD and, according to Hallowell and Ratey, the label might change again ([34,63]. Is it possible to recognize the profile, based on our research and knowledge, and let these children develop with their profile and abilities, before we find a need for a diagnosis? More knowledge is certainly needed, but the potential for sport as a positive developmental factor for children with ADHD symptoms is definitely promising.

This study has focused on investigating a cognitive profile of ADHD in young athletes and how it differs in different contexts (i.e., school and sport). There is an evident need for more studies concerning young individuals with the cognitive profile of ADHD and how they best develop a functional daily life in school and in leisure time. There is a need to learn more about how sport, with its physical exercise and stimulation, structure, consistent patterns of behavior and the positive reinforcement, can be useful in other areas such as school [38,64].

Looking at the strength of this study, although more comprehensive evaluation has been suggested for athletes seeking help [65], research shows the ASRS screener is a very good tool for clinical evaluation of ADHD [44], if made by trained clinical interviewers. In the current study the ASRS was administrated by trained clinical interviewers with experience in sport, Cognitive Behavioral Therapy (CBT) and ADHD. The participants were also assessed in two different settings (i.e., school and leisure/sport). An important limitation of the study was the limited information about the participant’s school performance (e.g., school grades) as an objective measure, which is suggested for future research. Furthermore, we did not control for school history, gender and school motivation. However, in the National sport system, the students are enrolled in regular school programs and they study in the same classes as non-sport students. Thus, they are less likely to be different on school-related variables. That said, future research still needs to take potential other variables into consideration such as gender, motivation and grades. Furthermore, in future studies the different aspects of ADHD are of interest, such as investigating hyperactivity and impulsivity in athletes and roles in sport participation.

In conclusion, in this study, we report that ADHD criteria are highly presented in the athlete group in the school environment and very low during leisure time compared to the non-athlete group. This supports the suggestion that ADHD symptoms might be more prevalent in an athlete population [66], but importantly, it varies with contexts. ADHD is mostly considered genetic [67], but studies show that the symptoms may be influenced by aspects such as stimulating physical activity and structure activity in a positive direction or can cause symptoms that cause difficulties [25,26,27]. Potentially these athletes are able to inhibit the symptoms and learned to show appropriate behavior with the right training [68], but this need to be further investigated in future research.

The ADHD criteria might turn into an advantage rather than a disadvantage for the athletes in their sport achievement [41]. Based on the hunter vs. farmer theory, criteria such as the ability to hyper focus, high energy, persistence, not giving up, and endurance are of particular interest as these are abilities that are necessary for achieving success in sports [16,22]. It is possible that, in combination with the structure in sport and the social competence ensuing from engagement in sports, this will become a positive ability often transferred to other areas, such as school life and academic achievement. Studies of highly successful entrepreneurs diagnosed with ADHD found that many of them described having higher energy levels than their peers and having the ability to stay hyper focused [35]. It is therefore suggested that we should look at the profile as a “diversity perspective” [69] and change from a model of “defect” to a model of “difference” and focus more on how the environmental conditions can influence the profile of ADHD [70]. Thus, the characteristics of a cognitive profile in athletes might possibly be an asset in the development of elite athletes instead of seen as a disadvantage [22,41].

## Figures and Tables

**Table 1:** Descriptive statistics for all study variables.

	Athletes		Controls	
	*M*	*SD*	*M*	*SD*
ASRS (school)	27.16	10.16	6.48	6.15
ASRS (leisure) AQ	8.76 14.30	6.16 4.05	6.09 14.24	5.71 4.91

Note: ASRS = the Adult ADHD Self-Report Scale. AQ = Autism Spectrum Questionnaire (AQ).

## Data Availability

The data presented in this study are available on request from the corresponding author.
